# Supplementary figures and images for: Physico-chemical characterization and topological analysis of pathogenesis-related proteins from Arabidopsis thaliana and Oryza sativa using in-silico approaches
Source: PLoS One. 2020 Sep 28;15(9):e0239836. doi: 10.1371/journal.pone.0239836 (PMC7521741; doi:10.1371/journal.pone.0239836)

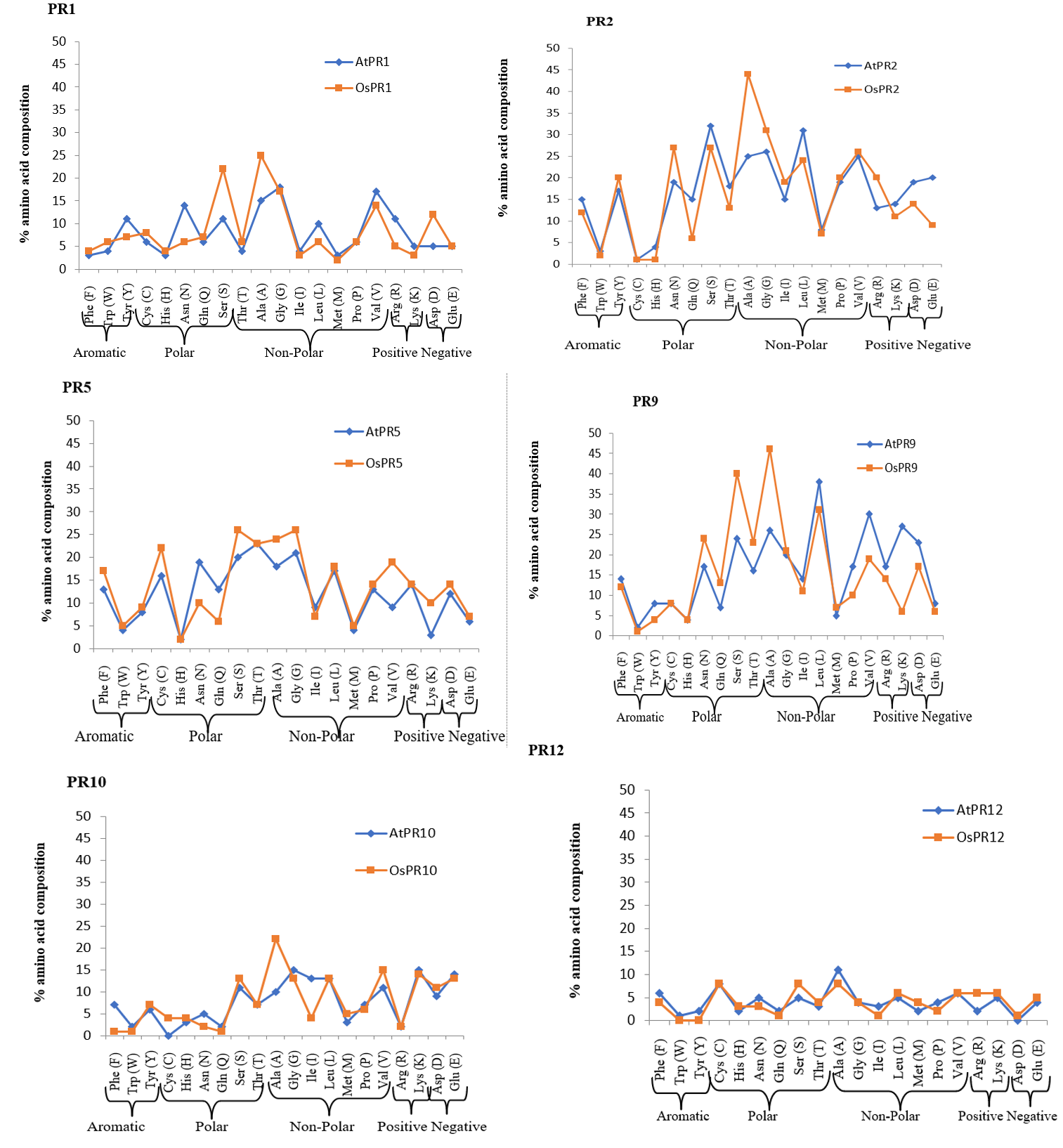

Supplement: S1 Fig — (TIF) [file pone.0239836.s001.tif]
